# Biocontrol capability of local *Metschnikowia* sp. isolates

**DOI:** 10.1007/s10482-019-01272-w

**Published:** 2019-05-20

**Authors:** Ewelina Pawlikowska, Steve A. James, Emilia Breierova, Hubert Antolak, Dorota Kregiel

**Affiliations:** 1 Institute of Fermentation Technology and Microbiology, Lodz University of Technology, Wolczanska 171/173, 90-924 Lodz, Poland; 2 Gut Microbes and Health, Quadram Institute Bioscience, Colney Lane, Norwich Research Park, Norwich, NR4 7UA UK; 3 Culture Collection of Yeasts (CCY), Institute of Chemistry, Slovak Academy of Sciences, Dúbravskácesta 9, 845 38 Bratislava, Slovakia

**Keywords:** Yeast, *Metschnikowia*, Screening, Stress, Adhesion, BCA

## Abstract

This study set out to isolate and identify epiphytic yeasts producing pulcherrimin, and to evaluate their potential as biological control agents (BCAs). We isolated *Metschnikowia* sp. strains from flowers and fruits collected in Poland. The plant material had been collected between April to September 2017 from two small orchards where traditional organic management is employed. We identified the essential phenotypic features of the yeast, including assimilation and enzymatic profiles, stress resistance, adhesion properties, and antimicrobial activity against various fungi involved in crop and/or food spoilage. Yeast screening was performed using YPD agar supplemented with chloramphenicol and Fe(III) ions. Taxonomic classification was determined by sequence analysis of the D1/D2 domains of the large subunit rRNA gene. The isolates were identified as *Metschnikowia andauensis* and *Metschnikowia sinensis.* The yeast isolates were further characterized based on their enzymatic and assimilation profiles, as well as their growth under various stress conditions. In addition, the hydrophobicity and adhesive abilities of the *Metschnikowia* isolates were determined using a MATH test and luminometry. Their antagonistic action against molds representing typical crop spoiling microflora was also evaluated. The assimilation profiles of the wild isolates were similar to those displayed by collection strains of *M. pulcherrima*. However, some of the isolates displayed more beneficial phenotypic properties, especially good growth under stress conditions. Several of the epiphytes grew well over a wider range of temperatures (8–30 °C) and pH levels (3–9), and additionally showed elevated tolerance to ethanol (8%), glucose (30%), and peroxides (50 mM). The hydrophobicity and adhesion of the yeast cells were strain- and surface-dependent. The tested yeasts showed potential for use as BCAs, with some exhibiting strong antagonism against molds belonging to the genera *Alternaria, Botrytis, Fusarium, Rhizopus,* and *Verticillium*, as well as against yeasts isolated as food spoilage microbiota.

## Introduction

Protection against losses caused by crop pests, plant diseases in particular, can play a critical role in improving food security worldwide. The protection of crops against plant diseases has an obvious role to play in the growing demand for food quality and quantity (Savary et al. 2012). Direct yield losses caused by plant pathogens reduce global agricultural productivity by between 20 and 50% (Kwasiborski et al. 2014; Savary et al. 2012). Molds are the most common post-harvest pathogens that affect fruits and vegetables. The prevention of these fungi, including mycotoxigenic species and strains, is considered a valid strategy to reduce the risks associated with fungal contamination of processed food and feed.

Chemical pesticides are typically used to prevent crop infections. The main purpose of conventional pesticide use is to minimize losses caused by crop diseases, and thus increase overall crop yields. It is believed that almost one third of all crops are treated with pesticides worldwide (Samsidar et al. 2018). Until recently, the use of synthetic pesticides for plant protection was thought to be fairly safe. However, nowadays the consensus has changed and it is widely recognized that the use of chemicals leads to three important issues, namely: (1) increased public concern regarding the contamination of plants with residues of synthetic fungicides and their effects on human health; (2) increased resistance of pathogens, and (3) environmental pollution (Abbey et al. 2018).

The European Food Safety Authority (EFSA) systematically performs acute dietary risk assessment for pesticide/food product combinations. Based on the latest scientific evidence, the EFSA has concluded that long-term dietary exposure to pesticides is unlikely to pose a health risk to consumers. Nevertheless, a number of non-approved substances have been found repeatedly in samples produced in the EU, which in some cases exceed the legal limits. Pesticides are also found occasionally in organic foods, which is not permitted for this type of product. For example, in the framework of the EU-coordinated control program (EUCP), 12,168 food samples were analyzed covering 165 pesticides in 11 food products. Although within the legally permitted levels, 46.0% of the samples were found to have quantifiable residues, while 0.9% of the samples were considered to be not compliant with the legal limits (EFSA 2018).

Currently, many companies whose activities have historically focused on the development and commercialization of chemical pesticides are now acquiring small companies that are already developing or have the potential to develop new microbe-based pesticides (Jensen et al. 2016). Beneficial microorganisms represent a large group of “good” agents which, due to their ability to interfere with the growth of plant pathogens and their positive effects on plants, are considered as potentially safe-to-use biopesticides (Glare et al. 2016; Le Cointe et al. 2016). The development of biopesticides has seen significant advances, primarily because of impressive progress in the isolation and characterization of novel strains of microorganisms which fulfill the main requirements for BCAs: strong inhibition of plant pathogens and the ability to be mass produced via cheap standard fermentation. However, although BCAs are now becoming more widely commercially available, they still constitute a small fraction of the total market for plant protection products (Regnault-Roger 2012). The main reason seems to be that biological control is viewed with caution and skepticism by many in the agricultural community. Biological control must be extremely efficient, in the range of 95–98%. Currently, this poses a significant challenge, and such levels are difficult to achieve on a reliable basis. It is essential that mass-produced products retain the properties of the initial lab-grown cultures. The formulation must maintain its species purity and the microbial cells their genetic stability, cell viability, and attributes as colonizers on fruit surfaces, as well as other aspects of their mechanism of action. The process must be cost effective, rely on industrial by-products as nutrients, and fermentation must be completed within 24–48 h. Therefore, it is critical to develop comprehensive knowledge of BCAs, including their antagonistic activities, growth under stress conditions, adherence to surfaces, as well as biofilm formation (Wisniewski et al. 2010).

Epiphytic yeasts seem a promising alternative to synthetic pesticides (Sipiczki 2006; Sui et al. 2015). Such yeasts are particularly valuable biological material, due to the fact they constitute a natural microflora of plants. Yeast strains originating from plants in the natural environment are well adapted and resistant to various factors, such as temperature and pH level, as well as osmotic and oxidative stresses. Epiphytic yeasts represent the largest component of the microbial community and have therefore adapted successfully to various specific ecological niches. Currently, the most promising yeasts appear to belong to *Metschnikowia pulcherrima*, *Trichosporon pullulans, Rhodotorula glutinis*, *Pichia membranifaciens, Issatchenkia orientalis, Candida* spp., *Cryptococcus laurentii* and *Pichia anomala* (Hu et al. 2017; Sui et al. 2015; El-Tarabily and Sivasithamparam 2006). These species have been used effectively as BCAs against a wide range of plant pathogens (Türkel et al. 2014). *Candida guilliermondii* versus *Aspergillus* spp., *M. pulcherrima* versus *A. niger,* and *Candida sake* versus *A. tubingensis* are three examples of yeast species that reduce grape colonization by mold pathogens (Sarrocco and Vannacci 2018).

Yeast strains belonging to *Metschnikowia* sp. are of particular interest (Kántor et al. 2015; Liu et al. 2017; Sipiczki 2006; Sisti and Savini 2014). In addition to the classical ways of action (i.e. competition for nutrients and space) and stress tolerance, the unique modes of biocontrol action employed by these yeasts are secretion of pulcherriminic acid and the ability to complex with Fe ions. Moreover, *Metschnikowia* sp. is able to secrete extracellular lytic enzymes, such as chitinase and glucosidases, which contribute to overall antifungal effects (Banani et al. 2015; Fia et al. 2005; Parafati et al. 2015; Saravanakumar et al. 2008). In turn, their metabolite pulcherriminic acid forms a chelate complex with iron ions. Therefore, the antagonistic action of *Metschnikowia* sp. is principally based on the depletion of iron, which is necessary for the growth of pathogens. Sipiczki (2006) showed that the antibacterial and antifungal activity of *M. pulcherrima* depends on the binding of iron in the growth medium. Hence, *M. pulcherrima* strains that produce high amounts of pulcherrimin are of great interest as growth inhibitors against pathogenic microorganisms (Kántor et al. 2015).

The aim of this study was to isolate and identify epiphytic yeasts producing pulcherrimin, and to evaluate their potential as BCAs. Their essential phenotypic features were determined, including assimilation and enzymatic profiles, stress resistance, adhesion properties and antimicrobial activity against various fungi involved in crop and/or food spoilage.

## Materials and methods

### Plant material

Flowers and fruits were collected between April and September 2017 in the Lodz Region, Poland (latitude 51°46′36″N; longitude 19°27′17″E) from two small orchards where traditional organic management was employed (Table 1). The samples were collected aseptically using sterile gloves and plastic bags and immediately stored for several hours in a refrigerator. All the samples were then processed.

**Table 1:** Plant material used in the study

No.	Plant material (variety)		Systematic name	Harvesting time
1	Fruits	Apple (Golden Delicious)	*Malus domestica* Borkh.	September 2017
2		Red grapes (Alden)	*Vitis vinifera* L.	September 2017
3		Raspberry (Heritage)	*Rubus idaeus* L.	September 2017
4		Red currant (Rosetta)	*Ribes spicatum*	July 2017
5		Strawberry (Senga Sengana)	*Fragaria *× *ananassa*	May 2017
6	Flowers	Strawberry (Senga Sengana)	*Fragaria *× *ananassa*	July 2017

### Collection strains of *Metschnikowia pulcherrima*

Five collections strains of *Metschnikowia pulcherrima* were used as reference material. Two strains of *M. pulcherrima*, NCYC747 and NCYC2321, were obtained from the National Collection of Yeast Cultures (Norwich, United Kingdom), while three strains, CCY 29-2-145, CCY 29-2-147, and CCY 29-2-149, were received from the Culture Collection of Yeasts (Bratislava, Slovakia). The strains were revived from stocks and sub-cultured on potato dextrose agar (PDA) slants [4 g/L potato infusion, 20 g/L glucose, 15 g/L agar] (Merck Millipore, Darmstadt, Germany) at 25 °C for 3 days. The yeast cultures were stored on YPD agar slants (Merck Millipore, Darmstadt, Germany) at 4 °C.

### Fungal strains

To evaluate the antagonistic activities of the yeast isolates, five mold strains originating from the Culture Collection of Microorganisms LOCK105 (Lodz, Poland) were used: *Alternaria alternata* LOCK409, *Botrytis cinerea* LOCK453, *Penicillium expansum* (syn. *P. glaucum*) LOCK535, *Rhizopus oryzae* LOCK547, and *Verticillium cinnabarinum* LOCK576. The molds were stored on YPD agar slants (Merck Millipore, Darmstadt, Germany) at 4 °C. They were preliminarily tested for pathogenicity on strawberry fruits. In addition, the wine strain *Saccharomyces cerevisiae* Tokay (LOCK203), yeasts *Wickerhamomyces anomalus* C1 (NCYC D5299), and *Dekkera bruxellensis* C2 (NCYC D5300), isolated from spoiled soft drinks in Poland, were used as test material (Kregiel et al. 2018).

### Screening of pulcherrimin-producing yeasts

A 10 g sample of fruit material was gently homogenized in 90 mL of sterile distilled water and incubated for 5 h at room temperature (20–22 °C). In the case of flowers, 10–15 inflorescences were processed. A 100 μL aliquot of each resulting homogenate was spread onto YGC agar plates supplemented with 0.05% (*w/v*) FeCl_3_ solution [5 g/L yeast extract, 20 g/L glucose, 0.1 g/L chloramphenicol, 20 g/L agar] (Merck Millipore, Darmstadt, Germany) and incubated at 25 °C for 3 days. From each sample, 10 typical dark red or brown coloured colonies were selected randomly. For the isolation of pure cultures, the spread plate method and YGC agar with 0.05% FeCl_3_ were used to obtain pure cultures of yeasts producing pulcherrimin. The incubation conditions were the same as those used for the screening procedure. The pure yeast cultures were stored on YPD agar slants (Merck Millipore, Darmstadt, Germany) at 4 °C.

### Identification of yeast isolates

Species identification of all wild yeast isolates was performed by the National Collection of Yeast Cultures (Norwich, United Kingdom) by sequencing the D1/D2 variable domains of the larger RNA subunit gene (LSU). The variable D1 and D2 domains of the LSU rRNA gene were PCR-amplified as a single DNA fragment directly from whole yeast cell suspensions, following the procedure and PCR parameters described by Kregiel et al. (2018). The yeast LSU D1/D2 domain was amplified and sequenced using the conserved fungal primers NL1 (GCATATCAATAAGCGGAGGAAAAG) and NL4 (GGTCCGTGTTTCAAGACGG) (O’Donnell 1993). The amplified LSU D1/D2 products were separated using 1.0% agarose electrophoresis, then purified and concentrated using QIAquick PCR purification spin columns (QIAGEN) according to the manufacturer’s instructions. A NanoDrop 1000 Spectrophotometer (Thermo Scientific) was used to measure the DNA concentration, and the samples were sequenced by a commercial sequencing facility (Eurofins MWG Operon). Sequence traces were edited manually, and consensus sequences were generated using the program SEQMAN, version 11 (DNASTAR). The LSU D1/D2 sequence of each yeast strain was compared with sequences held in the EMBL/GenBank sequence databases. The sequences were deposited in the GenBank database with assigned accession numbers. The yeast isolates have been deposited in the Culture Collection of Microorganisms LOCK105 (Lodz, Poland, WFCC No. 598, WDCM No. 105).

### Yeast cultivation and testing

In order to assess the morphological features of the yeast isolates, they were cultivated in YPD broth (Merck Millipore, Darmstadt, Germany) for 2 days on a Heidolph Titramax 1000 rotary shaker (Heidolph, Schwabach, Germany) at 125 rpm. Pulcherrimin production was evaluated in YPD broth with 0.05% FeCl_3_. The yeast cells and pulcherrimin were observed using an OLYMPUS BX41 light microscope (Olympus, Shinjuku, Tokio, Japan) connected to a DP72 digital camera. In order to determine growth profiles under varying environmental conditions, including temperature (2–55 °C), pH (2–9), peroxide (0–50 mM), glucose (0.5–30% w/v), and ethanol concentration (1–8% w/v), YPD medium was also used. Inoculation of the culture media was carried out using standardized suspensions of the tested yeasts [~ 1°McF]. The intensity of yeast growth was measured using a DEN-1densitometer (Grant Instruments, Cambridge, UK) and expressed on the McFarland scale [°McF].

### Assimilation profiles

The assimilation profile of each yeast isolate was determined using the API 20 C AUX identification system (BioMérieux, Lyon, France), following the manufacturer’s instructions. The resulting assimilation profiles for the wild isolates were compared to those determined previously for collection *M. pulcherrima* strains.

### Enzymatic fingerprinting

The enzymatic profiles of the yeast isolates were determined using the API ZYM test (BioMérieux, Lyon, France). Inoculation and evaluation were carried out based on the manufacturer’s instructions and recommendations. The profiles of the isolates were compared to those obtained for collection *M. pulcherrima* strains.

### Adhesion abilities

White glass slides (G) were used as the reference hydrophilic material (76 × 26 mm, Star Frost, Knittel Glass, Braunschweig, Germany) and polypropylene (PP) as the reference hydrophobic surface (76 × 26 mm, Paccor Packaging, Skierniewice, Poland). The values for the contact angles of the reference materials were determined as 44.2 ± 4.3° and 92 ± 4.7°, respectively (Antolak et al. 2018).

The minimal culture medium [3 g/L (NH_4_)_2_SO_4_, 1 g/L KH_2_PO_4_, 1 g/L K_2_HPO_4_, 0.5 g/L MgSO_4_ × 7H_2_O, 1 g/L yeast extract, 10 g/L glucose] was sterilized at 121 °C. Into 50 mL Erlenmeyer flasks was poured 25 mL of the medium, into which sterile glass carriers were placed vertically in such a way that part of the carrier was immersed while the rest was outside the liquid. The inoculum was standardized to obtain a cell concentration in the culture medium of approximately 10^2^–10^3^ CFU/mL at the beginning of the experiment. The samples were incubated at 25 °C on a laboratory shaker (125 rpm) for 7 days.

Cell adhesion to the carriers was analyzed by luminometry and scanning electron microscopy (SEM). For luminometric tests, the carrier was removed from the culture medium, rinsed with sterile distilled water and swabbed using free-ATP sampling pens (Merck Millipore, Darmstadt, Germany). The measurements were reported in relative light units per square centimeter (RLU/cm^3^) using a HY-LiTE^®^ 2 luminometer (Merck Millipore, Darmstadt, Germany) (Kregiel 2013b). For SEM studies, a Jeol JSH 5500 LV microscope was used in high vacuum mode at an accelerating voltage of 10 kV. The samples were coated with a fine layer of gold (approximately 20 nm thick) using an ion-coating JEOL JFC 1200 apparatus (Fortuniak et al. 2018).

### Hydrophobicity

The ability of yeast cells to adhere to hydrocarbons was determined as a measure of their hydrophobicity (MATH test), according to the method described by Kregiel (2013a). Planktonic yeast cells were cultivated in the minimal medium and harvested by centrifugation at 5000 rpm for 5 min at 4 °C. They were then washed twice in PBS and finally resuspended in the same buffer. The cell suspensions were adjusted to an A_0_ value of approximately 5.0 at 540 nm (Spekol 220, Zeiss, Germany), before 2 mL of the yeast suspensions were added to 0.4 mL of xylene (OBR PR, Poland) and vortexed for 60 s. The two phases were allowed to separate for 10 min at 25 °C. The aqueous phase was removed carefully and the A value at 540 nm was measured once more. A decrease in the absorbance of the aqueous phase was taken as a measure of cell surface hydrophobicity (H), calculated using the following formula: H = [(A_0_ − A)/A_0_] × 100%, where A_0_ and A are the absorbance before and after extraction with xylene, respectively. The yeast cells were classified in terms of their percentage affinity to xylene using the following scale: < 10% hydrophilic, 10–29% medium hydrophilic, 30–55% medium hydrophobic, > 55% highly hydrophobic.

### Antagonism in vitro

The antagonistic effects of the yeast isolates against spore germination and the hyphal growth of different mold species were tested using the cross method with some modifications (Balouiri et al. 2016). A loopful of spores from fully sporulated mold strains was collected aseptically and resuspended in 1 mL of sterile saline. The obtained spore suspensions (~ 10^6^ × mL^−1^, 100 µL) were spread evenly on the YPD agar plates. Then yeast cell suspensions (~ 10^7^ × mL^−1^, 2 μL) were dropped on the plates in duplicate, and the plates were incubated at 25 °C for 7 days. Inhibition zones were measured (mm) at the end of the incubation period. All experiments on the antagonistic effects of yeast strains were performed in triplicate.

### Efficacy: preliminary studies on soft fruits

Fresh strawberries, harvested in a Polish orchard and grown according to organic management practices, were washed in sterile saline (0.85% *w/v*) and drained at room temperature. Each fruit portion (~ 5 g) was exposed to spray treatment with 2 mL of a yeast suspension (~ 10^7^ cells × mL^−1^) of a *Metschnikowia* sp. strain. After 1 h, 2 mL of a spore suspension of grey mold *B. cinerea* (~ 10^6^ spores × mL^−1^) was pipetted onto the fruit. An inoculated control was also performed using 2 mL of conidial suspension. The fruits were packed randomly in commercial plastic boxes and stored at 10 °C for 3 weeks. A trained panel of 10 individuals was used to evaluate their sensorial properties before and after storage. The samples after washing with tap water were assessed in terms of color and flavor. Each attribute was scored on the following 3-point hedonic scale: 1 = acceptable; 2 = perceptibly altered but still acceptable; 3 = unacceptable. The taste, odor, and color typical of the fruits, as exemplified by frozen samples that had been thawed prior to the sensory evaluation, were regarded as acceptable. All experiments were performed in duplicate (Nowak et al. 2016).

### Statistics

The mean results taken from three independent experiments were calculated. Comparisons between the mean values were performed using the One-Way ANOVA test (STATISTICA 10, TIBCO Software Inc., Palo Alto, CA, USA) followed by a Tukey’s multiple comparison test. Assimilation and enzymatic profiles were obtained from API 20 C AUX and API ZYM tests results (BioMérieux, Lyon, France). Assimilation tests and enzymatic fingerprinting were visualized by hierarchical clustering using ClustVis (https://biit.cs.ut.ee/clustvis/), a web tool for presenting multivariate data. Other yeast profiles were created with Plotly (https://plot.ly/#/), a free web tool for data visualization (Antolak et al. 2018, 2019).

## Results and discussion

### Screening and identification

**Fig. 1:**
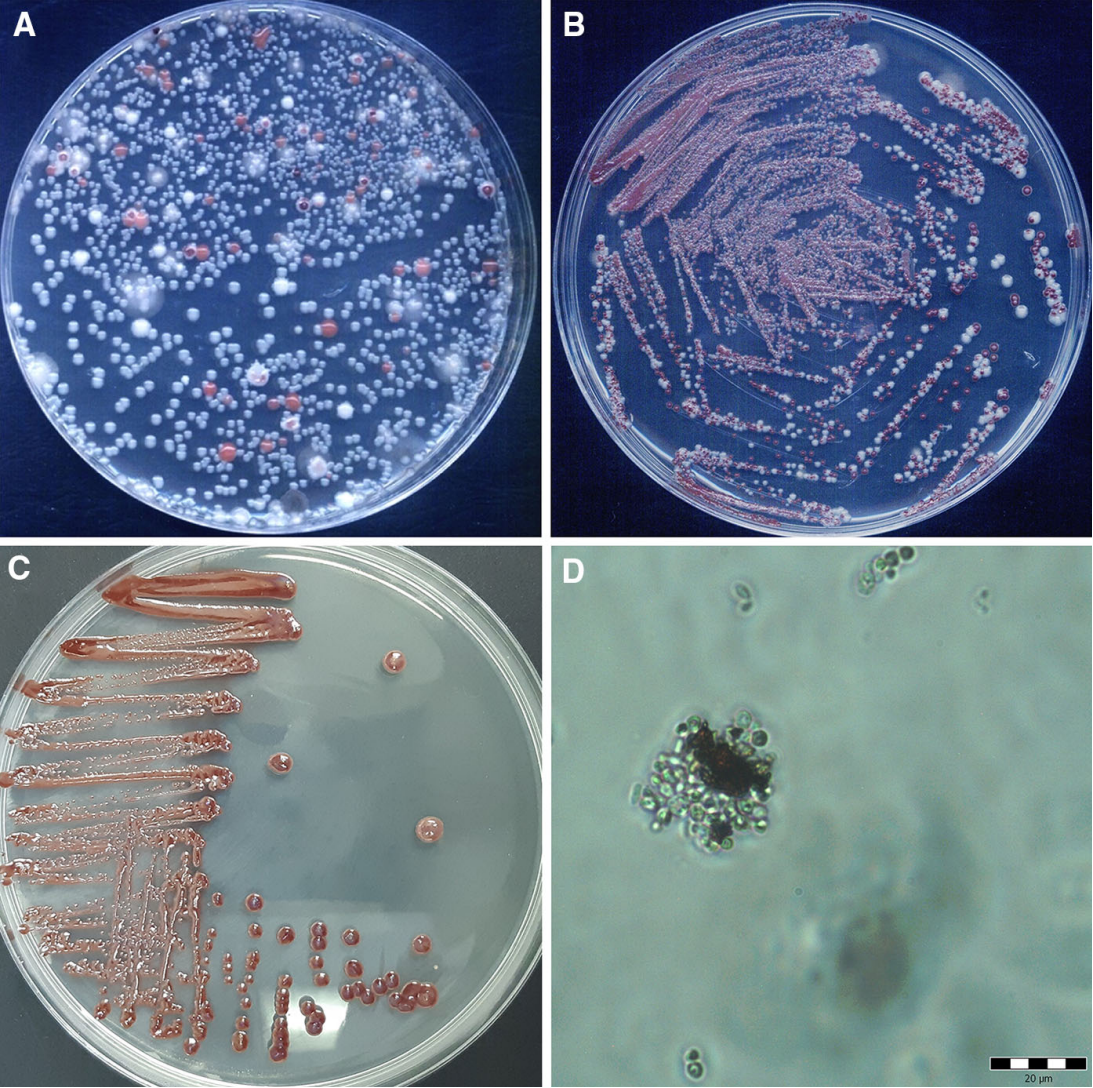
Screening (**a**) and isolation (**b**, **c**) of yeasts on YGC agar medium with Fe(III). **d** Yeast cells harvested from YPD broth with Fe(III) visible using a light microscope

The yeast screening method we employed, using YGC agar supplemented with Fe(III) ions, allowed us to obtain characteristic brown or red colonies (Fig. 1a–c). From the total plant material, 48 characteristic clones were isolated, and from these the 10 yeast isolates that had formed the darkest colonies (labelled D1 to D10) were selected for further studies. The streak plate procedure was conducted several times on YPD agar to ensure that each isolate was a monoculture. Microscopic observations were made to determine the morphology of each monoculture (Fig. 1d). The isolate cell size typically ranged from 2 to 12 μm in length and from 2 to 10 μm in width. All the yeast isolates were reproduced vegetatively by budding, with some cells forming groups while others remained separate. In yeast cultures obtained from YPD broth with added Fe(III) ions, visible amorphous red inclusions accumulated in both the cells and the growth medium. This standard procedure for recovering *Metschnikowia* sp. isolates based on pulcherrimin formation has been used in numerous studies (Kántor et al. 2015; Oro et al. 2014; Pretscher et al. 2018; Sipiczki 2006; Türkel and Ener 2009). It is well-documented that the antimicrobial activity of *M. pulcherrima* strains depends on pulcherrimin pigment production, which in turn inhibits the growth of sensitive microorganisms by iron sequestration (Sipiczki 2006). The yeast isolates that produced large amounts of pulcherrimin and thus grew as dark brown colonies in the presence of Fe(III) ions were therefore selected, since they were of the greatest interest as potential inhibitors of pathogenic microorganisms.

All the yeast isolates were identified by ribosomal DNA (rDNA) sequencing. Each yeast monoculture was identified to species level by sequencing the D1/D2 domains of the large subunit rRNA gene. The 10 selected yeast isolates (D1–D10) were taxonomically classified as either *Metschnikowia andauensis* or *Metschnikowia sinensis* (Table 2). A similar identification procedure is used by the NCYC Collection for various yeasts. This methodology has also been applied to identify *Wickerhamomyces* spp., *Candida* spp., *Kazachstania* spp. and *Dekkera* spp. isolates (James et al. 2013, 2014, 2015; Kregiel et al. 2018). However, it is worth noting that each isolate displayed a notable level of sequence variation in comparison to the respective species type strain. Modern yeast taxonomy is based on phylogenetic analysis of conserved DNA regions or protein sequences. By far the most frequently used sequences are those of the D1 and D2 domains of the large subunit (LSU, 26S) rDNA. Most researchers opt to use this DNA marker for species identification of yeast, because the rDNA repeats within an individual genome generally have identical sequences (due to sequence homogenization). However, in a recent study Sipiczki et al. (2013) discovered that the rDNA arrays of certain *Metschnikowia* strains were in fact not fully homogenized. While seemingly rare, rDNA heterogeneity has been observed in yeasts, perhaps most notably in *Saccharomyces cerevisiae* (James et al. 2009) and most recently in *Kluyveromyces marxianus* (Perpetuini et al. 2018).

**Table 2:** Results of molecular identification of yeast isolates

No.	Strain symbol	Origin		Species	GenBank accession number
1	D1	Fruits	Apple	*M. sinensis*	MK612094
2	D2			*M. andauensis*	MK612095
3	D3		Raspberry	*M. sinensis*	MK612096
4	D4			*M. andauensis*	MK612097
5	D5		Grape	*M. andauensis*	MK612098
6	D6			*M. andauensis*	MK612099
7	D7		Red currant	*M. andauensis*	MK612100
8	D8			*M. andauensis*	MK612101
9	D9		Strawberry	*M. sinensis*	MK612102
10	D10	Flower	Strawberry	*M. sinensis*	MK612103

Previous studies of ascomycetous yeasts have revealed that strains displaying more than 1% sequence variation in the D1/D2 domains usually belong to separate species (Kurtzman and Robnett 1998). The intragenomic D1/D2 sequence diversity of *Metschnikowia* sp. strains D1–D10 exceeded this intraspecies diversity. Phylogenetic analysis of the 26S rDNA D1/D2 domain sequence showed that *M. andauensis* and *M. sinensis* are clustered in a clade together with *M. fructicola*, *M. pulcherrima* and *M. chrysoperlae* (Xue et al. 2006). Kurtzman and co-workers hypothesize that hybridization contributes to the intragenomic diversity of *Metschnikowia* (Kurtzman et al. 2018). This phenomenon has been demonstrated experimentally by Sipiczki and co-workers (Sipiczki et al. 2018). Thus, the intra-strain sequence variation we observed in our isolates may indicate that they possess rDNA arrays of hybrid origin, i.e. rDNA from two parental *Metschnikowia* sp. A possible explanation for the observed intra-strain (intragenomic) sequence variation observed in isolates D1–D10 may therefore be intraspecies and/or interspecies hybridizations. Recently, significant divergences have been reported in strains belonging to *Clavispora lusitaniae* and *Metschnikowia bowlesiae* (Lachance and Fedor 2014; Durrens et al. 2017).

Sipiczki et al. (2018) found a high degree of sequence diversity in the D1/D2 domains of two pulcherrimin-producing *Metschnikowia* species. The sequences of the internal transcribed spacers (ITS) were also not homogenized and differed from each other. This high intragenomic diversity makes the D1/D2 domains and the ITS spacers unsuitable for barcoding. Therefore, the taxonomic position of the strains previously assigned to them requires revision. Because of this peculiarity of pulcherrimin-producing *Metschnikowia* strains, precise taxonomic identification is practically impossible. To emphasize this fact, the present study uses symbols for strains D1–D10 belonging to *Metschnikowia* sp.

### Assimilation profiles

**Fig. 2:**
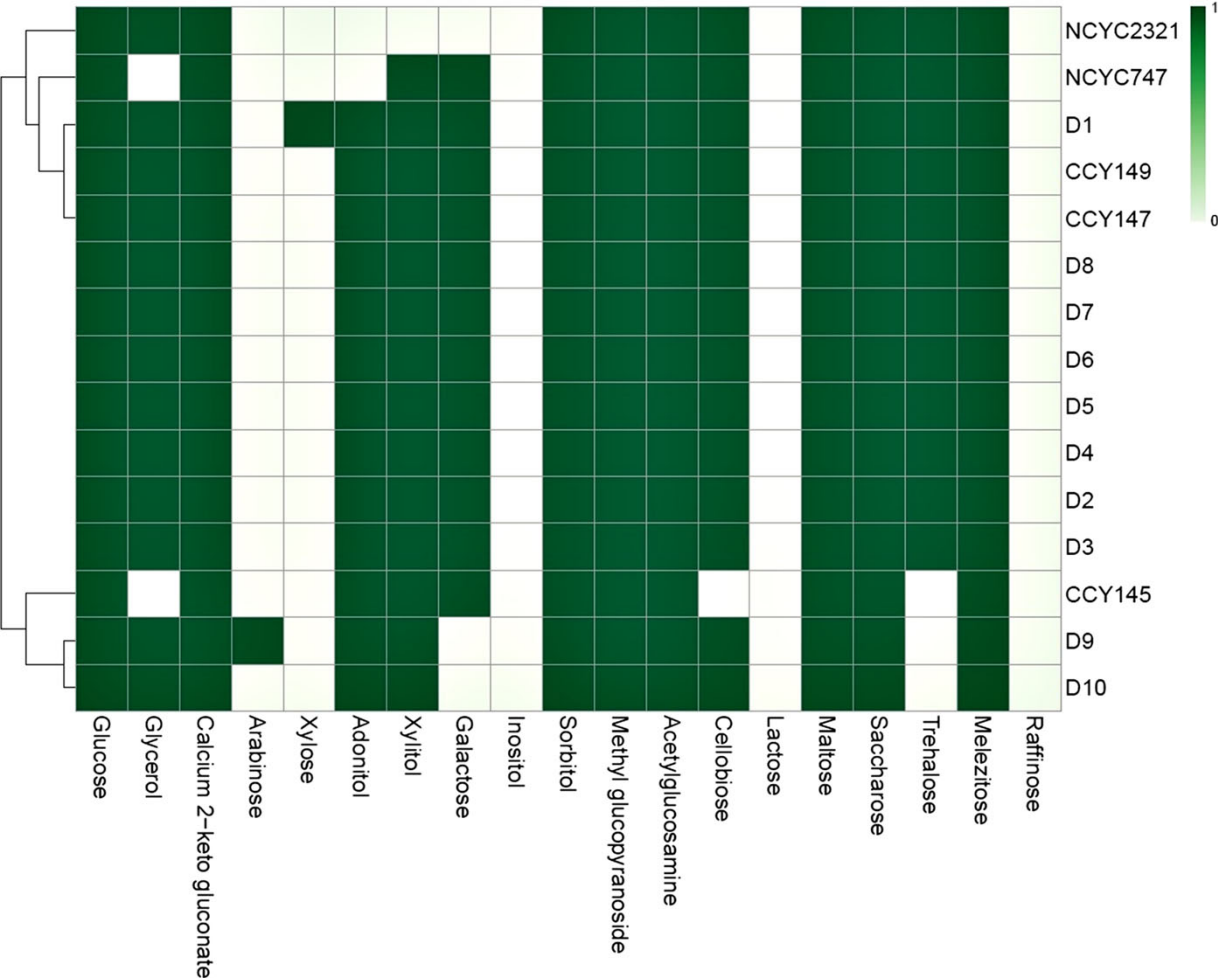
Assimilation profiles. Hierarchical clustering of positive/negative results. Distance measure: Euclidean. Clustering algorithm: Ward. Each row represents an individual strain, while each column represents a carbon source

Of the carbon compounds used to characterize yeasts, two sugars, sucrose and L-sorbose, are usually utilized by most *Metschnikowia* sp., whereas the assimilation of inulin, raffinose, lactose, starch, rhamnose, L- and D-arabinose, methanol, erythritol, galactitol, inositol, and glucuronic acid is rather rare. Other carbon compounds, such as β-glucosides, ethanol, glycerol, mannitol, glucitol, succinic and 2-ketogluconic acids, and N-acetylglucosamine may also be assimilated, but there is variation between strains (Lachance et al. 2016). All the tested yeasts assimilated all the carbon sources except raffinose, lactose, and inositol (Fig. 2). However, strong differences were noticeable between the collection strains and the set of isolates used in our study. For example, isolates D1 and D9 are able to assimilate xylose and arabinose, respectively. Narrow assimilation profiles were observed in the collection strains *M. pulcherrima* NCYC2321 and NCYC747, but the broadest profile was seen for isolate D1. The use of the ClustVis program allowed us to group the tested yeast strains into three main clusters: the first and third clusters contained both collection strains and epiphytes with very diverse profiles, while the second cluster was composed of isolates D2 to D8, which had the same assimilation profile.

### Enzymatic fingerprinting

**Fig. 3:**
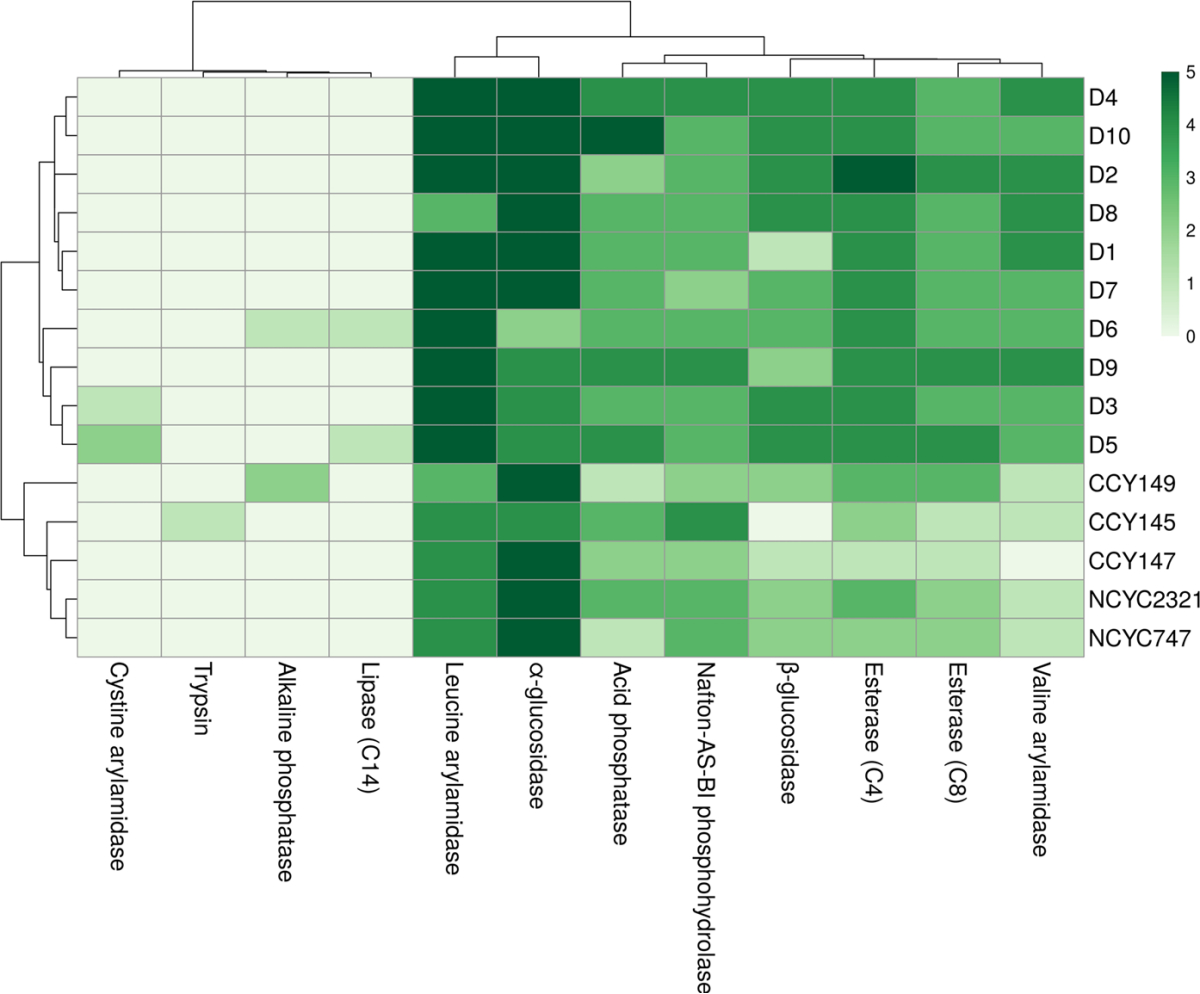
Enzymatic fingerprinting. Hierarchical clustering of positive (1–5) and negative (0) results. Distance measure: Euclidean. Clustering algorithm: Ward. Each row represents an individual strain, while each column represents an enzyme. (For interpretation of the references to color in this figure legend, the reader is referred to the web version of this article). (Color figure online)

The enzyme systems for fungal cell lysis are usually a mixture of several different enzymes, including one or more β-1,3- and β-1,6-glucanases, proteases, mannanases, and chitinases, all acting synergistically to lyse the cell wall. It is worth noting that the activity of one group of enzymes may influence that of the second. For example, α- and β-glucosidase activities may stimulate the action of α- and β-glucanases on cell-wall components (Banani et al. 2015; Pretscher et al. 2018). Enzymatic profiles obtained from the API ZYM system indicate that all the tested strains showed α-glucosidase and leucine arylamidase activities (Fig. 3). The results for α-chymotrypsin, α-galactosidase, β-galactosidase, β-glucuronidase, N-acetyl-β-glucosaminidase, α-mannosidase, and α-fucosidase were either negative or very weak for all the tested strains. They were therefore excluded from the comparative analysis of *Metschnikowia* strains. In general, a distinct difference was observed between the collection strains and isolates D1–D10. The epiphytic yeasts showed higher enzymatic activities for esterases, valine arylamidase, acid phosphatase, β-glucosidase and naphtol-AS-BI-phosphohydrolase.

The use of hierarchical clustering allowed us to group the yeast strains into 3 main clusters. The first contained all the collection strains, the second comprised isolates D3, D5, D6, and D9, while the third contained epiphytes D1, D2, D4, D7, D8, and D10, which displayed the strongest enzymatic activity. The wide spectrum of enzymatic action may also play an important role in biocontrol mechanisms. The hydrolytic activity of yeasts can play an important role in both their colonization and invasion (Staniszewska et al. 2013). It is worth noting the activity of leucine arylamidase in all the tested strains. This enzyme is a member of the metalo-peptidase group, which removes the N-terminal l-leucine from peptide substrates. Leucine arylamidase activity provides leucine molecules needed for the formation of pulcherrimine acid, and finally pulcherrimin (Kántor et al. 2015; Sipiczki 2006).

### Stress resistance

Microorganisms found in natural environments and under industrial conditions are exposed to high fluctuations, caused by variable weather conditions as well as crop storage methods. Thus, to be successful biological control agents antagonists need to show effective mechanisms to cope with the plethora of abiotic stresses to which they will be exposed. Further studies are necessary on the effects of numerous environmental factors on potential BCA, especially to assess the viability of yeast antagonists (Sui et al. 2015).

**Fig. 4:**
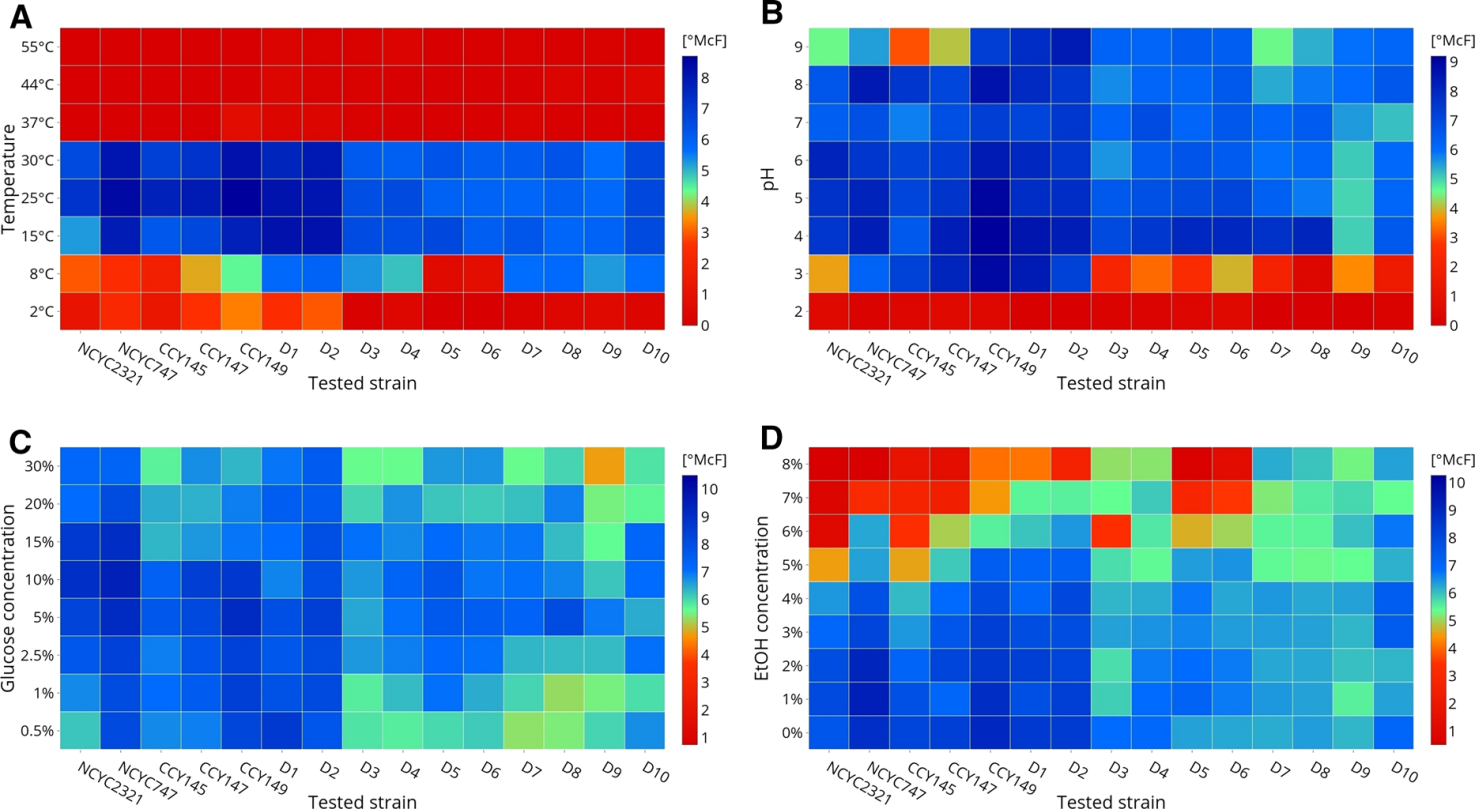
Growth profiles under stress conditions. Each row represents a tested parameter, while each column represents a tested strain. The blue color corresponds to the highest values, while the red color corresponds to the lowest. (For interpretation of the references to color in this figure legend, the reader is referred to the web version of this article). (Color figure online)

Figure 4a presents temperature profiles for the growth of the tested yeast strains in culture media incubated over a wide range of temperatures, from 2 to 55 °C. All of the tested strains were able to grow at between 8 and 30 °C, but the best growth was recorded at temperatures between 15 and 30 °C. According to Lachance et al. (2016), growth at the elevated temperature of 37 °C is characteristic for species belonging to the *M. lunata* clade. However, isolates from tropical climate may show good growth at higher temperatures. For example, an epiphytic isolate of *M. persimmonesis* collected in South Korea was found to be able to grow at 40 °C (Kang et al. 2017). Furthermore, it is possible to improve the thermal resistance of some yeast strains. For example, in studies by Liu et al. (2011), mild heat shock pretreatment at 40 °C was found to improve the heat tolerance of *M. fructicola*, resulting in it being able to grow at 45 °C.

In the present study, the *M. pulcherrima* collection strains NCYC747, CCY 29-2-147, and CCY 29-2-149, as well as isolates D1 and D2, were all able to grow at 2 °C. Vegetables and fruits are usually stored at low temperatures, in order to extend their commercial shelf life and availability. Therefore, the ability of yeasts to colonize and develop on a host (the commodity) at low temperatures is an important biocontrol feature (Sangorrín et al. 2014; Sui et al. 2015).

Figure 4b shows the results of yeast growth in culture medium with different pH values, ranging from 2 to 9. All the tested strains were characterized by the ability to grow over a broad range of pH, from 3 to 9. The most significant yeast growth occurred when the pH values of the medium ranged from 4 to 8. Similar results were obtained in a previous study for *M. pulcherrima* (Spadaro et al. 2010), whereas a strain of *M. persimmonesis* isolated from Korean persimmon showed higher optimal pH 6 (Kang et al. 2017). Good growth at pH 3 was particularly evident for collection strains NCYC747, CCY 29-2-145, CCY 29-2-147, and CCY 29-2-149, as well as two epiphytes isolated from apples (D1 and D2). According to the literature, stress-adapted yeasts growing well at pH 4 can effectively inhibit apple rots caused by the mold *P. expansum* (Liu et al. 2012). As in the case of temperature stress, it is also possible to improve the resistance of yeasts to low pH. Wang et al. (2014) noted that low pH tolerance in the basidiomycetous yeast *Rhodosporidium paludigenum* may be improved by incubation in media with pH levels ranging from 4.0 to 5.5. This pretreatment resulted in a higher growth rate on apples and thus greater biocontrol efficacy.

The ability to withstand osmotic stress (dehydration, freezing, and osmotic agents) is another trait that can make yeasts suitable for use as BCAs (Sui et al. 2015). Figure 4c shows the growth of yeasts in the presence of glucose at concentrations ranging from 0.5 to 30% (*w/v*). Despite the fact that all the tested strains were characterized by relatively high osmotic tolerance, two collection strains (NCYC2321 and NCYC747) as well as some isolates (e.g. D1, D2) showed a greater ability to withstand osmotic stress in comparison to the other strains tested. Osmotolerance—growth in the presence of 50% glucose—varies widely in *Metschnikowia* sp. (Lachance et al. 2016). As microbial growth at high sugar concentration is advantageous for biocontrol activity, yeasts isolated from high-osmotic environments may also be a good source of new BCAs (Liu et al. 2013).

Figure 4d shows the ethanol tolerance of the tested yeast strains. The results indicate that all the tested strains showed growth in the presence of 5% (*v/v*) ethanol. However, many of our isolates were able to grow in the presence of 8% (*v/v*) alcohol. Yeasts belonging to *M. pulcherrima* with low fermentation capacity (about 4% *v/v*) have been described as also having rather low ethanol tolerance (3–6% *v/v*) (Aponte and Blaiotta 2016; Lachance et al. 2016). However, in a study conducted by Barbosa et al. (2018), some *M. pulcherrima* strains were found to be able to tolerate up to 9% *v/v* ethanol. This confirms the results of our studies. Ethanol tolerance may be important feature of BCAs in wine-making. Molds are unable to grow in wine, but their effect on wine quality is due to grape damage and the production of specific metabolites in the early stages of fermentation. The selection and use of ‘autochthonous yeasts’ as biocontrol agents in early fermentation may assist the prevention of fungal spoilage (Mills et al. 2002).

**Fig. 5:**
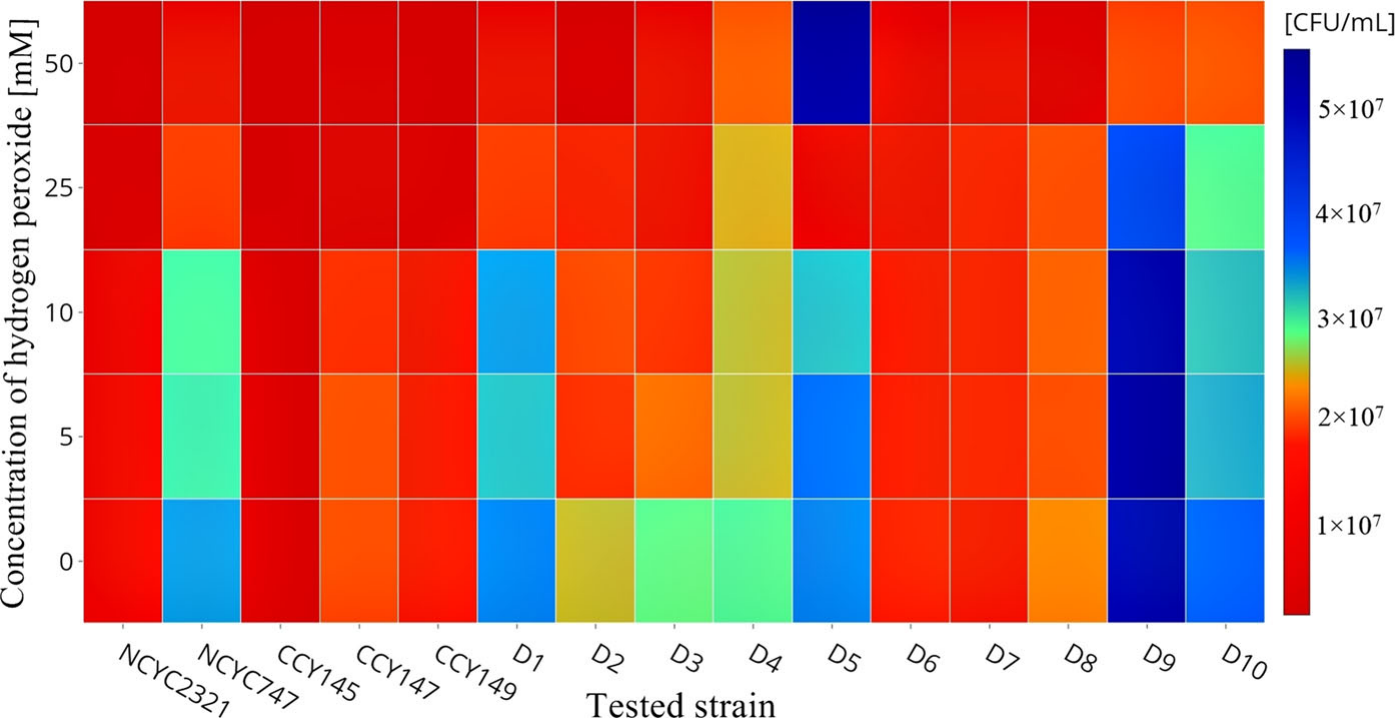
Growth in the presence of peroxides (H_2_O_2_). Each row represents a tested parameter, while each column represents a tested strain. The blue color corresponds to the highest values, while the red color corresponds to the lowest. (For interpretation of the references to color in this figure legend, the reader is referred to the web version of this article). (Color figure online)

It has been suggested that reactive oxygen species (ROS) signalling is a component in the mode of action of yeast antagonists in biocontrol systems (Chi et al. 2015). Antagonistic yeasts may serve as elicitors, triggering ROS signalling in host tissue and leading to the activation of host defences (Sui et al. 2015). This postulate is supported by studies analysing the molecular basis of yeast-fruit interactions. Biocontrol agents must be able to tolerate ROS-derived oxidative stress, which can affect their viability and efficacy. Yeasts with higher resistance to ROS-generated oxidative stress exhibit greater colonization and better biocontrol efficacy (Castoria et al. 2003). Liu et al. (2011) examined the responses of strains belonging to *Metschnikowia* sp. to oxidative stress. They found that *M. fructicola* was the most tolerant species, surviving well in 200 mM H_2_O_2_. Li et al. (2014) report that the survival of *Pichia caribbica* decreased significantly as the concentration of H_2_O_2_ was increased from 5 to 20 mM. Figure 5 shows the growth of the tested strains of *Metschnikowia* sp. in different concentrations of H_2_O_2_. The controls were samples without the addition of peroxide. In general, the isolates showed greater resistance to hydrogen peroxide, especially strains D4, D5, D9, and D10, which survived at a level of 2 × 10^7^ CFU/mL in the presence of 50 mM/mL H_2_O_2_. On the other hand, the greatest sensitivity to the test compound was shown by collection strains NCYC2321, CCY 29-2-145, and CCY 29-2-147, as well as by isolates D2 and D8.

### Hydrophobicity and adhesion abilities

The ability to form biofilms is as an effective mechanism of action for some BCAs. One of the most important factors is the initial attachment of biofilm, since cell adhesion is a necessary first step in biofilm formation (Liu et al. 2014). In the natural environment, crop surfaces are frequently wetted by rainfall, dewfall, fog, or cloud mist. Therefore, the special hydrophobicity/hydrophilicity of BCA cells may contribute to better adhesion properties to the surface of crops and reduce the overall leaching of yeast cells (Abdel-Kader et al. 2012; Krasowska and Sigler 2014).

**Fig. 6:**
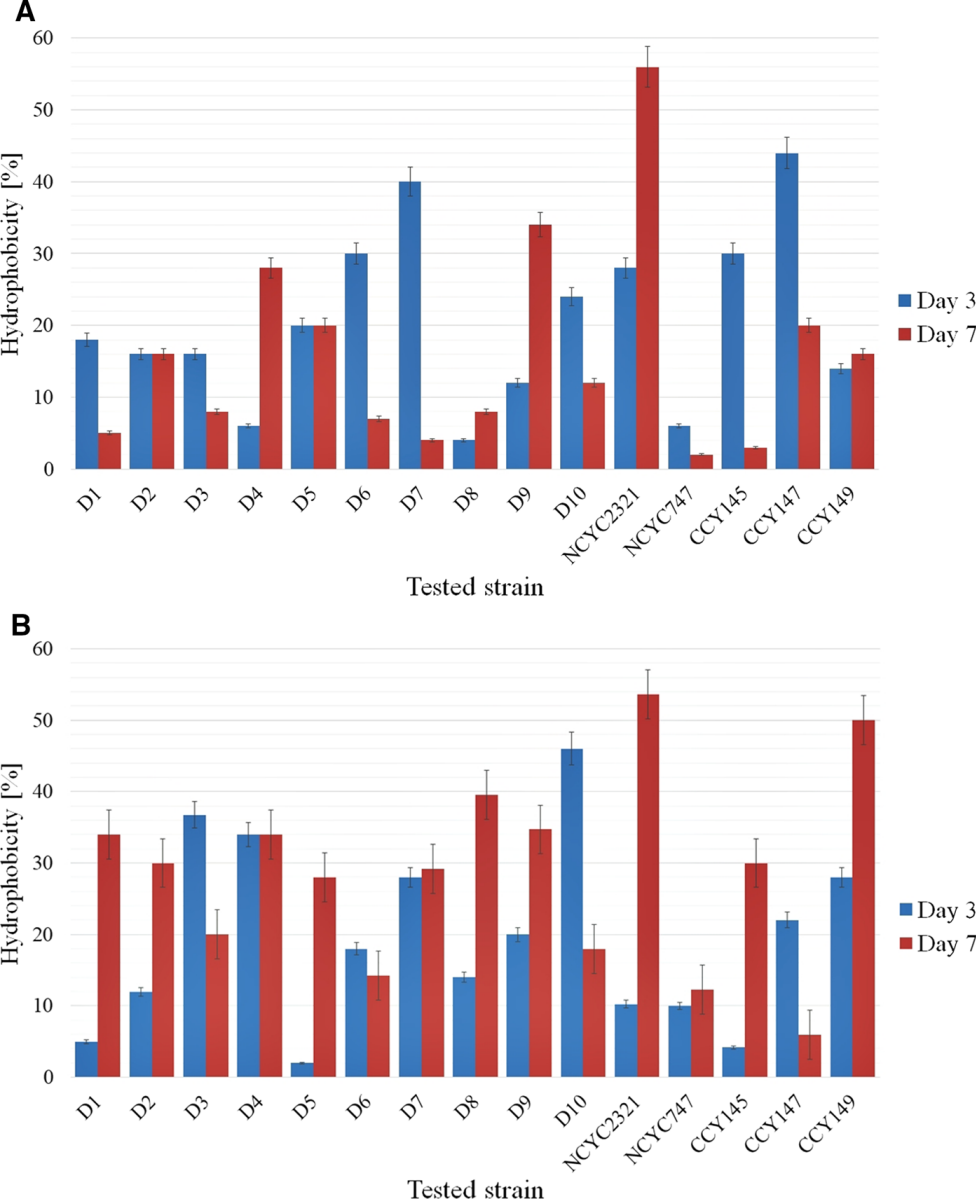
Hydrophobic properties of yeast cells harvested from two culture media: **a** YPD broth, **b** minimal medium

We studied the hydrophobic properties of yeast cells harvested from two differed culture media: minimal medium and rich YPD broth. In general, the yeast cells harvested from the rich culture medium showed more hydrophilic properties (Fig. 6a) than those grown in the minimal medium (Fig. 6b). The only exceptions were the strain NCYC2321, which displayed hydrophobicity in both culture media, and strain CCY29-2-147, which showed hydrophilic properties in the minimal medium. Similar results were obtained by Rodríguez et al. (2018), who noted that the *M. pulcherrima* strain showed rather hydrophilic properties.

**Fig. 7:**
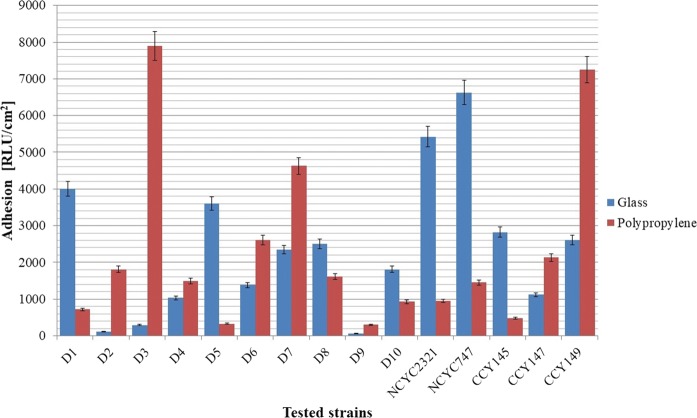
Cell adhesion to glass and polypropylene surfaces (RLU/cm^2^)

Over millions of years of evolution, plants have developed a wide diversity of surfaces adapted to their specific environments. The structural basis of hydrophobic and hydrophilic plant surfaces is variable. It may be based on smooth surfaces, but also surface structuring, such as water-absorbing hairs, porous and sponge-like structures, or rough convex surface structures. In this study, we used well-defined abiotic carriers. Yeast cell adhesion to two types of substrate, namely glass (hydrophilic) and polypropylene (hydrophobic), was analyzed by luminometry and scanning electron microscopy. According to the literature, luminometry is an efficient and reliable way to determine microbial attachment to surfaces (Kregiel 2013b). The approach is based on bacterial ATP quantification, and can be applied to detect not only adhered cells, but also their extracellular secretions. Figure 7 presents the results of yeast adhesion to a glass surface (RLU/cm^2^) in minimal medium, where most strains displayed higher level of hydrophobicity. The levels of cell adhesion to glass and polypropylene varied, ranging from 70 to 6600 RLU/cm^2^ and from 250 to 7900 RLU/cm^2^, respectively. The luminometric results were both strain and surface dependent. Slightly higher results were obtained for the plastic surface and for half of the strains tested, regardless of their origin.

**Fig. 8:**
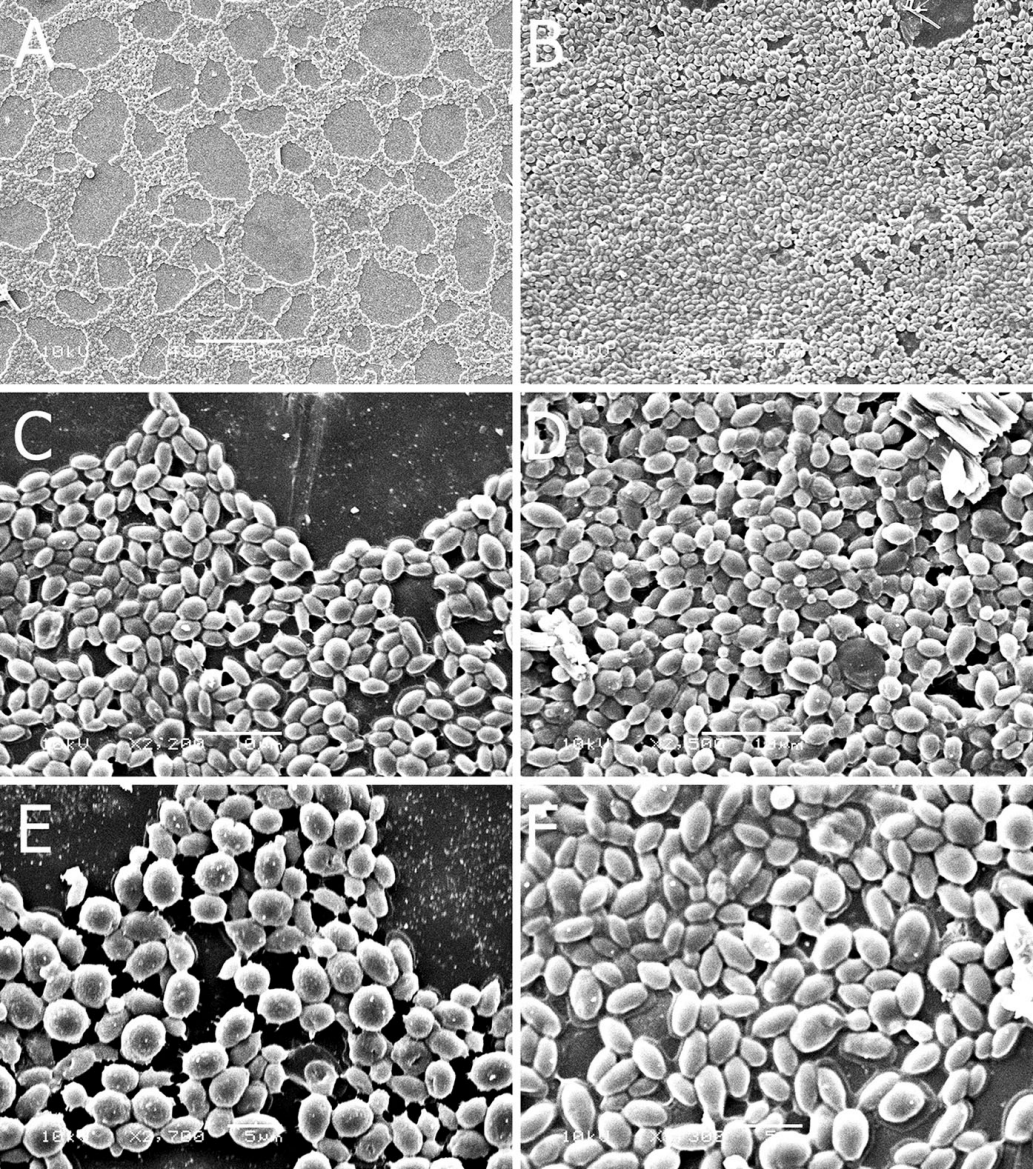
Cell adhesion to the glass and polyethylene surfaces observed in SEM

A qualitative analysis of cell adhesion was also conducted, based on SEM. Figure 8 shows images of the glass (Fig. 8a, c, e) and polypropylene (Fig. 8b, d, f) after 5-day incubation with isolate D3 in minimal medium. In general, microscopic observations confirmed the luminometric results. The best adhesion was observed on the hydrophobic plastic material, resulting in surface coverage of approximately 80–90% of the total area. In addition, it was observed under high magnification that the attached yeast cells were covered with an extracellular substance which could also be detected luminometrically (Fig. 8e, f). The good adhesion of the tested strains of *Metschnikowia* sp. to both hydrophobic and hydrophilic surfaces suggests that they may be able to colonize plant surfaces effectively. This feature reinforces the conclusion that *Metschnikowia* sp. is a promising source of BCA.

### Antagonism in vitro

**Fig. 9:**
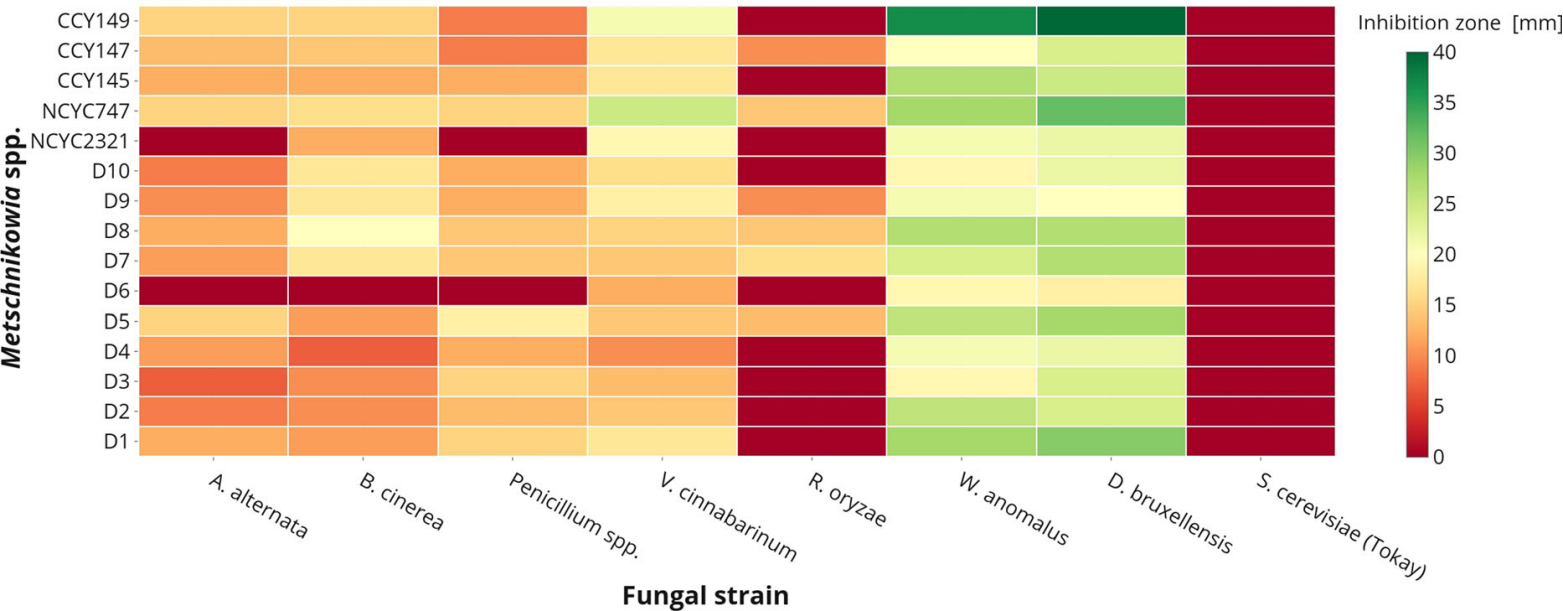
Antagonism of *Metschnikowia* sp. strains. Each row represents the tested *Metschnikowia* strain, while each column represents a tested fungal microorganism. The green color corresponds to the highest values in the inhibition zone, while the red color corresponds to lack of inhibition. (For interpretation of the references to color in this figure legend, the reader is referred to the web version of this article). (Color figure online)

Figure 9 presents the inhibitory activity of the *Metschnikowia* strains against 5 pathogenic mold strains. The tested yeast strains inhibited various fungal pathogens. However, the halo of inhibition was strain-dependent. Inhibition has also been observed for the yeasts *W. anomalus* and *D. bruxellensis*, which are often isolated as spoilage microbiota in foods and beverages (Kregiel et al. 2018), although not for the *S. cerevisiae* Tokay wine strain. It is worth noting that *W. anomalus* has likewise been suggested as a possible biocontrol yeast (Kurtzman 2011; Zhang et al. 2019).

It has been reported that iron sequestration inhibits conidial germination and hyphae growth, by inducing autolysis at the hyphal tips (Sipiczki 2006). Moreover, mutants that do not form pulcherrimin also lack antifungal activity. Therefore, in *Metschnikowia* yeast the contribution of other factors (e.g. wall lytic enzymes) to antifungal activity seems to be very minor compared to that of iron complexing.

### Efficacy on soft fruits

**Fig. 10:**
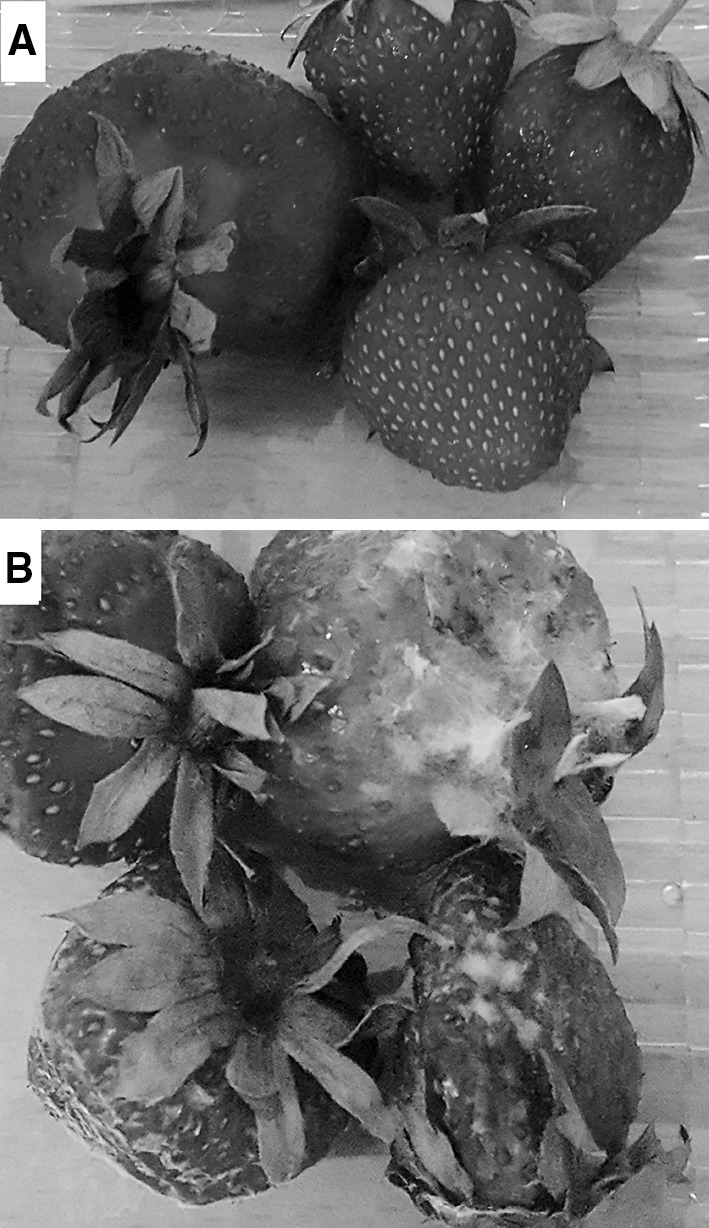
Strawberry fruits after 3-week storage at 8 °C. **a** Fruits treated by grey mold and isolate D2; **b** fruits treated with grey mold (control sample)

The influence of *Metschnikowia* sp. strains on the organoleptic qualities of strawberries was evaluated after treatment with a *Botrytis cinerea* conidial suspension. Of the numerous pathogens that cause fruit diseases in strawberries, the gray mold *B. cinereal* is the most widespread globally. In addition, this mold is known for its innate resistance to fungicides, which is due to its high genetic variability, profuse production of spores, and multiple cycles of spore production and disease development (Koike and Bolda 2016). Therefore, this fungal pathogen was selected for use in our preliminary studies. The addition of yeast suspensions did not have any negative effect on the sensory attributes of the strawberries. Initially, all the tested fruits were acceptable. After 3 weeks of refrigerated storage, the control samples treated with *B. cinerea* showed a loss of organoleptic quality, and the growth of grey mold was also visible. In contrast, all the other strawberry samples treated with both *B. cinerea* and isolate D2 remained visually acceptable after a 3-week period of storage at 8 °C (Table 3, Fig. 10a). Control samples (contaminated by mold, without the addition of yeast cells) obtained a score of 3.0 (Fig. 10b). It is worth noting that the fruit surfaces treated with *Metschnikowia* sp. took on a characteristic intense red color, and the flavor either remained specific or an additional pleasant floral aroma was perceptible.

**Table 3:** Sensory analysis of strawberry fruits stored at 8 °C

Storage time (weeks)	Yeast strain	Color	Flavor
0	NCYC2321	1.0 ± 0.0	1.0 ± 0.0
	NCYC747	1.0 ± 0.0	1.0 ± 0.0
	CCY145	1.0 ± 0.0	1.0 ± 0.0
	CCY147	1.0 ± 0.0	1.0 ± 0.0
	CCY149	1.0 ± 0.0	1.0 ± 0.0
	D1	1.0 ± 0.0	1.0 ± 0.0
	D2	1.0 ± 0.0	1.0 ± 0.0
	D3	1.0 ± 0.0	1.0 ± 0.0
	D4	1.0 ± 0.0	1.0 ± 0.0
	D5	1.0 ± 0.0	1.0 ± 0.0
	D6	1.0 ± 0.0	1.0 ± 0.0
	D7	1.0 ± 0.0	1.0 ± 0.0
	D8	1.0 ± 0.0	1.0 ± 0.0
	D9	1.0 ± 0.0	1.0 ± 0.0
	D10	1.0 ± 0.0	1.0 ± 0.0
	Control	1.0 ± 0.0	1.0 ± 0.0
3	NCYC2321	1.3 ± 0.1	1.0 ± 0.0
	NCYC747	1.6 ± 0.1	1.0 ± 0.0
	CCY145	1.5 ± 0.1	1.0 ± 0.0
	CCY147	1.0 ± 0.0	1.0 ± 0.0
	CCY149	1.6 ± 0.2	1.0 ± 0.0
	D1	1.2 ± 0.1	1.0 ± 0.0
	D2	1.0 ± 0.0	1.4 ± 0.2
	D3	1.4 ± 0.3	1.0 ± 0.0
	D4	1.3 ± 0.3	1.0 ± 0.0
	D5	1.3 ± 0.2	1.0 ± 0.0
	D6	1.4 ± 0.3	1.2 ± 0.2
	D7	1.3 ± 0.1	1.0 ± 0.0
	D8	1.2 ± 0.3	1.3 ± 0.2
	D9	1.3 ± 0.1	1.0 ± 0.0
	D10	1.2 ± 0.3	1.0 ± 0.0
	Control	3.0 ± 0.0	3.0 ± 0.0

Hedonic scale: 1 = acceptable; 2 = perceptibly altered but still acceptable; 3 = unacceptable

Quantitative variables are expressed as means and standard deviations. “–” not evaluated

## Conclusions

This study forms part of research aimed at identifying new effective BASs for large-scale applications. It is worth noting that commercialized biofungicides containing yeast strains *M. fruticola* have already been developed, such as the bioproduct “Shemer” (AgroNews 2010). Formal EU approval has been granted to yeast-based biofungicides using *M fructicola* strain NRRL Y-27328, following a favorable vote by EU member states (Commission Implementing Regulation (EU) 2018/1915). The *Metschnikowia* isolates tested in our study show promising features as potential BCAs. They inhibited common fungi, widespread in the world as plant pathogens and causative agents of food spoilage. They were characterized by various phenotypical properties, and most grew well under stressful conditions, as did the culture collection strains. The isolates showed a slightly wider assimilation spectrum than the collection strains. However, for effective implementation under field conditions, the use of BCAs requires an integrative approach, including additional practices that can decrease the incidence or severity of disease. These methods may include appropriate crop rotation and management of crop residues, the application of organic amendments and the use of new technologies, such as the biological disinfestation of soils.
